# A Network of Genes, Genetic Disorders, and Brain Areas

**DOI:** 10.1371/journal.pone.0020907

**Published:** 2011-06-10

**Authors:** Satoru Hayasaka, Christina E. Hugenschmidt, Paul J. Laurienti

**Affiliations:** 1 Department of Biostatistical Sciences, Wake Forest University School of Medicine, Winston-Salem, North Carolina, United States of America; 2 Department of Radiology, Wake Forest University School of Medicine, Winston-Salem, North Carolina, United States of America; 3 Center for Genomics and Personalized Medicine Research, Wake Forest University School of Medicine, Winston-Salem, North Carolina, United States of America; National Institutes of Health, United States of America

## Abstract

The network-based approach has been used to describe the relationship among genes and various phenotypes, producing a network describing complex biological relationships. Such networks can be constructed by aggregating previously reported associations in the literature from various databases. In this work, we applied the network-based approach to investigate how different brain areas are associated to genetic disorders and genes. In particular, a tripartite network with genes, genetic diseases, and brain areas was constructed based on the associations among them reported in the literature through text mining. In the resulting network, a disproportionately large number of gene-disease and disease-brain associations were attributed to a small subset of genes, diseases, and brain areas. Furthermore, a small number of brain areas were found to be associated with a large number of the same genes and diseases. These core brain regions encompassed the areas identified by the previous genome-wide association studies, and suggest potential areas of focus in the future imaging genetics research. The approach outlined in this work demonstrates the utility of the network-based approach in studying genetic effects on the brain.

## Introduction

The human diseasome network by Goh et al. [1] presented a stunning visual overview of the complex relationship between genes and diseases. Without focusing on a particular disease or gene, they were able to demonstrate how different diseases are related through common genes. The network also showed clustering of diseases according to their disease classes (e.g., bone, cancer, cardiovascular, etc.), indicating some level of common or shared genetic influences affecting the same tissues, organs, or biological systems. This amazing network of human diseases and genes outlines the potential of using network science in studying biological systems. In fact, genetic networks have been used as prediction tools to identify complex relationships among genes [2]–[4]. Such networks can also be augmented with other types of data, including protein-protein interaction [5]–[8], phenotype information [5],[8],[9], gene-disease associations [1], [7]–[12], and other “omics” data [3], [5], [8]. Construction of such networks can be accomplished by text-mining of the literature [1], [8]–[10], [12] or by mining existing data sets [4]–[6], [8], [11].

Organizing existing data and analysis results for such networks, needless to say, is far more economical compared toactually conducting a genomics study. A typical study for genetic associations may require data collection and assays, which can be time consuming and labor intensive. In addition, statistical analyses of such data often require special techniques to account for unique characteristics of the data, such as the family structure, massive multiple comparisons, population biases, or non-normality. On the other hand, the network-based approach requires far fewerresources in terms of costs and manpower involved. Most of the effort is focused on culling multiple databases and organizing findings in the form of a network. The network-based approach enables combining of data from multiple populations together, thus allowing investigators to focus on a large number of genes and/or phenotypes simultaneously.Such a network may represent consolidated results from multiple separate studies, but each of the studies does not have to be as extensive as, for example, a genome-wide association study (GWAS).

Some of the disease classes in the human diseasome network [1] pertain to the brain. This is not surprising especially when we consider the increasing number of studies linking genetic factors to the brain. For example, the heritability maps on the human [13] and the baboon [14] showed regional differences in heritability in various brain areas. Moreover, recent advances in brain imaging technologies have identified associations between some genes and neuroimaging-derived phenotypes [15], [16] of various types, including the brain structure [17]–[23], the brain function [18], [19], [24]–[27], and the brain connectivity [28]–[32]. More recently, whole-brain GWASon schizophrenia [33] and on Alzheimer's disease [34]–[36] demonstrated the ability to localize associations between genetic markers and brain areas simultaneously. Despite the large amount of data involved in a whole-brain GWAS, however, such a study can only focus on one disease or condition at a time. In other words, typical genetic analyses focus on only one of the nodes in the human diseasome network [1] at a time. Although such studies are valuable, they are unable to address questions related to overall genetic influences on the brain. For example, common genes regulating the brain may also be related to multiple genetic disorders. Or, perhaps diseases affecting similar areas of the brain may be associated with the same genes. To appreciate an overview of genetic influences and associations on various brain areas, an approach akin to Goh et al.'s [1] would be ideal. To accomplish this, we connected various brain areas to the human diseasome network. This was done by connecting genetic diseases in the diseasome network and various brain areas that are known to be associated with those diseases. Such disease-brain connections were made based on text mining of the literature in the PubMed database (http://www.ncbi.nlm.nih.gov/pubmed) [37]. This process produced a tripartite network of genes, genetic disorders, and brain areas. The characteristics of the resulting network were examined. In particular, highly connected genes, genetic disorders, and brain areas were identified. Moreover, we investigated whether different brain areas were affected by the same set of diseases or were associated with the same set of genes.

## Results

### Tripartite Graph

**Figure 1:**
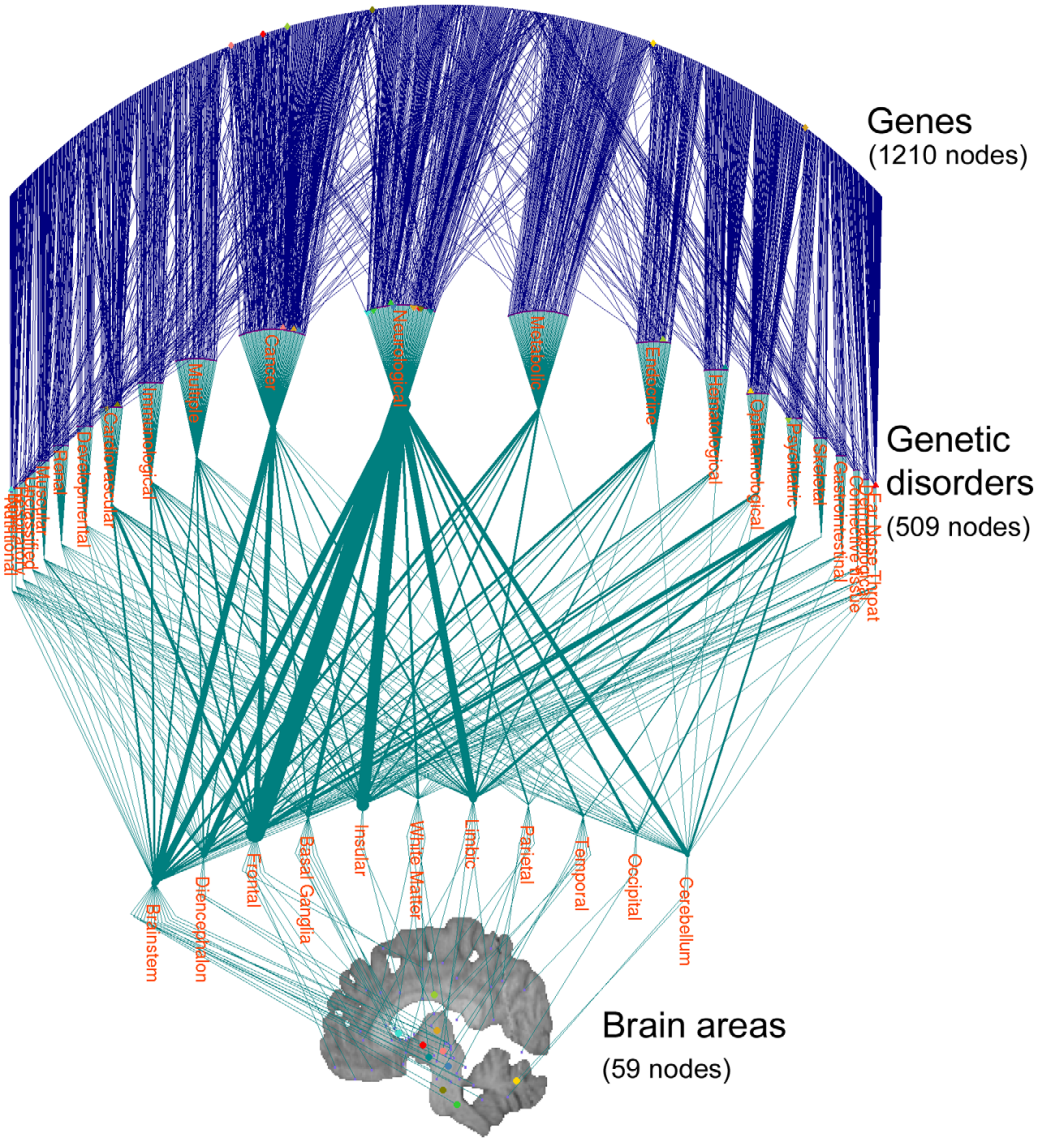
Gene-disease-brain area tripartite graph. The three layers are genes (1210 nodes), genetic disorders (509 nodes), and brain areas (59 nodes). Genetic disorders are grouped by disease types, and brain areas are grouped by lobes and large anatomical divisions. The thickness of the lines connecting disease classes and anatomical divisions are proportional to the number of connections bundled between the two groups of nodes.

Figure 1 shows the complete tripartite graph of genes, genetic disorders, and brain areas. To facilitate the presentation, the disease nodes were grouped into disease classes and the brain areas were grouped by larger anatomical divisions. The thickness of the lines connecting disease classes and anatomical divisions are proportional to the number of connections bundled between the two groups of nodes. From Figure 1, it can be seen that connections between the diseases and the brain areas are unevenly distributed, with the connections between neurological diseases and the frontal lobe being the most prominent.

### Degree Distributions

**Figure 2:**
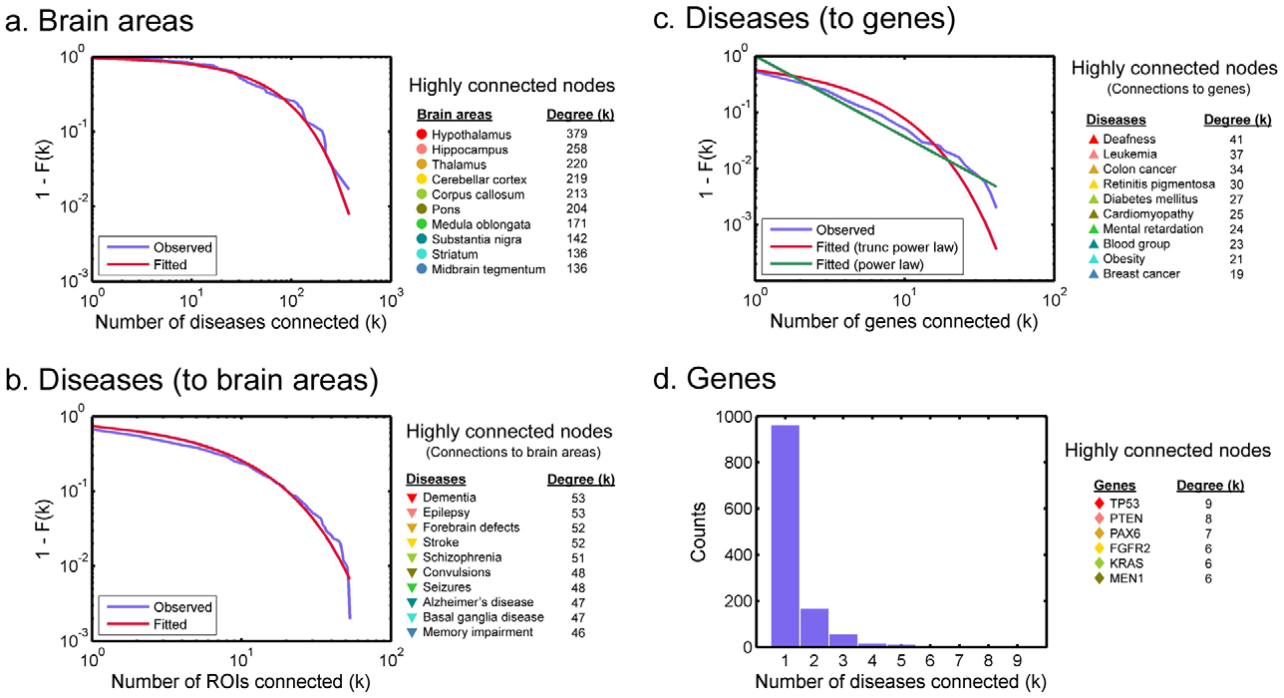
Degree distribution plots of the gene-disease-brain tripartite network. Degree distributions of brain areas (a), diseases to brain areas (b), diseases to genes (c), and genes (d) are shown. For the degree distributions for brain areas and diseases (a)–(c), the complementary cumulative distribution (1−*F*(*k*)) is plotted, whereas the actual frequencies are plotted for genes (d). Panel (d) is plotted as a bar graph since the range of degrees is limited and does not span a single order of magnitude. The best-fit exponentially truncated power law curves are also plotted for (a)–(c). In addition, the best-fit power law curve is also shown in (c). Highly connected nodes corresponding to each degree distribution are also listed, along with the markers corresponding to those nodes indicated in Figure 1.

The distributions of the number of connections at each node, or degree denoted by *k*, in the three layers of the tripartite graph were examined, and tremendous heterogeneity in the degree was observed. The degree distribution of the brain area nodes can be seen in Figure 2a. The figure shows the complementary cumulative distribution, or 1 minus the cumulative distribution function (CDF) 1−*F*(*k*). The distribution closely follows an exponentially truncated power law distribution (p = 0.78, Kolmogorov-Smirnov (KS) test), a long-tailed distribution indicating a few highly connected nodes witha large number of connections (*k*>200). This is in contrast with the vast majority of other nodes with just a few dozen connections; the median of the degree distribution is 32, and 75% of edges are connected to the top30% highest degree nodes. Connections originating from the disease nodes were also heterogeneously distributed. A small number of diseases affect a large number of brain areas extensively (see Figure 2b) and a small number of the diseases are associated with a large number of genes (see Figure 2c). Plotted on a log-log scale, both distributions exhibited characteristics of long-tail distributions, spanning over multiple orders of magnitude with very few high-degree nodes at the tail. In both distributions, 70% or more edges are connected to the top 30% highest degree nodes. However, bothobserved distributionsdid not fit exponentially truncated power law distributions or power law distributions according to KS tests (p<0.0001 in all cases). By visual inspection, the degree distribution for connections originating from diseases to brain areas exhibited an accelerated decay near the tail (Figure 2b), whereas the distribution for connections originating from diseases to genes seemed follow a straight line on a log-log scale (Figure 2c). This discrepancy may be due to the difference in the abundance of gene nodes and brain area nodes. While each disease node may connect to any of 1210 gene nodes, each disease node can connect to only up to 59 brain areas. Such a small number of available brain nodes may inhibit the occurrence of a node with an extremely large degree, resulting in a truncation in the degree distribution. Alternatively, this discrepancy in the shape of the distributions may be because the brain-disease system is inherently differently organized than the disease-gene system.Interestingly, there was not an overlap between the diseases highly connected to brain areas and the diseases highly connected to genes. This may be because not all genetic disorders have a strong neurological component. Connections originating from genes were also heterogeneously distributed. While the majority of genes were associated with only a single disease, there were a few genes associated with 6 or more diseases (see Figure 2d).

### Common Disease Network and Common Gene Network

**Figure 3:**
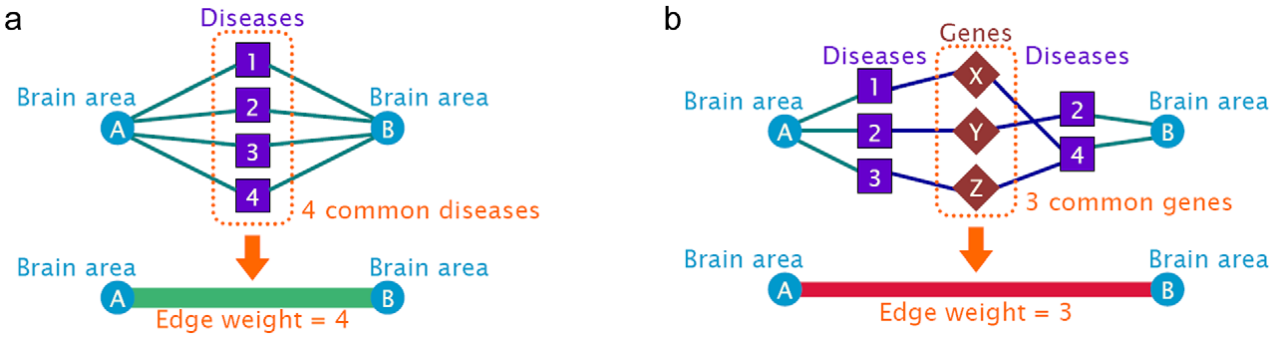
A schematic of forming connections for the common disease and gene networks. A connection is formed according to common diseases (a) and common genes (b) shared between two brain areas. The number of shared diseases or genes is used as the edge weight between two brain areas.

**Figure 4:**
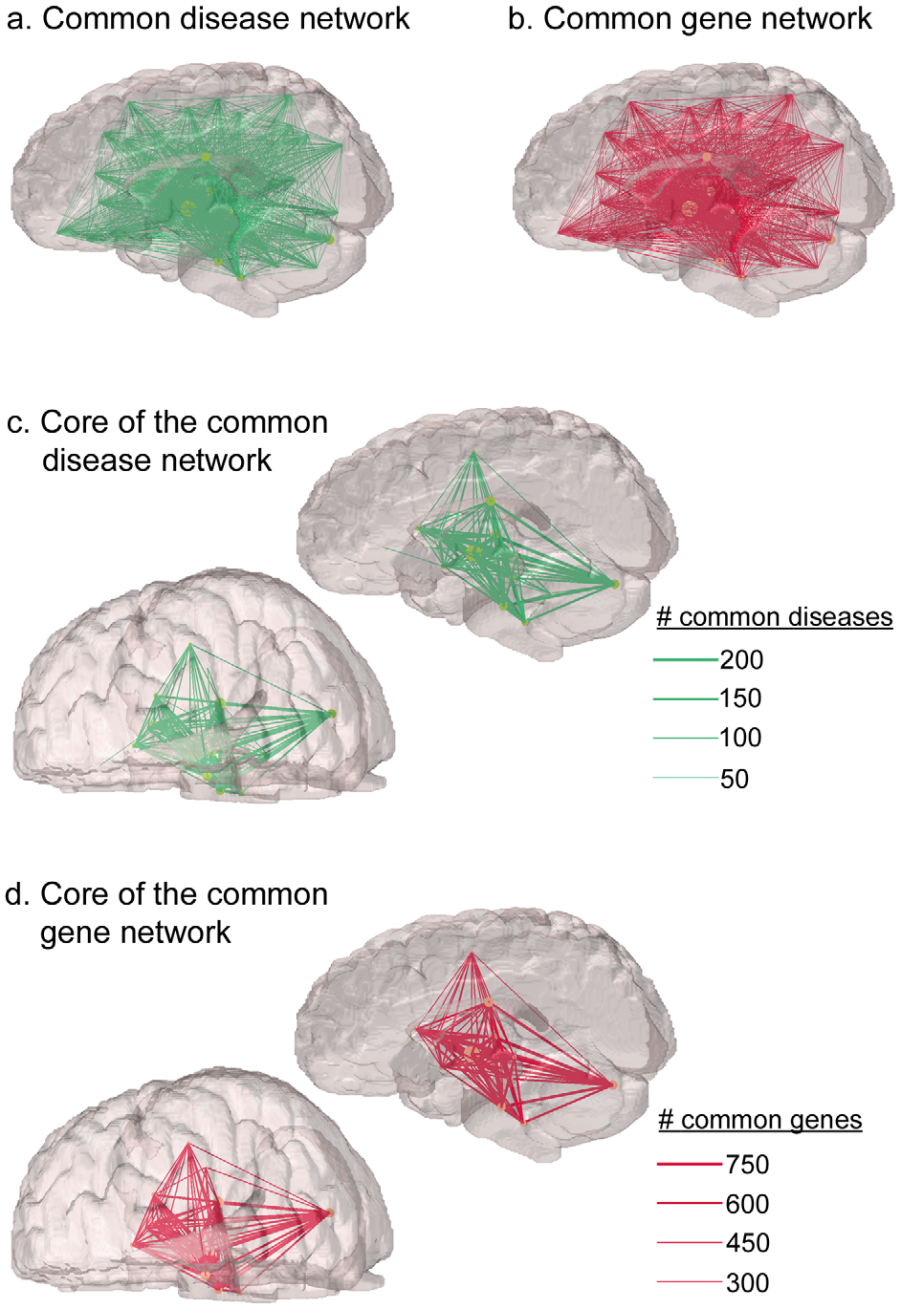
The common disease network and the common gene network. The full common disease network (a) and the full common gene network (b) show that most brain areas are connected to each other. However, the core of the common disease network (c) and the core of the common gene network (d), the networks with only the edges with top 10% connection weights, show that a small number of brain areas share a large number of diseases or genes in common. Moreover, both common networks consist of similar nodes possibly due to the fact that connections in the common gene network are mediated by disease nodes.

**Figure 5:**
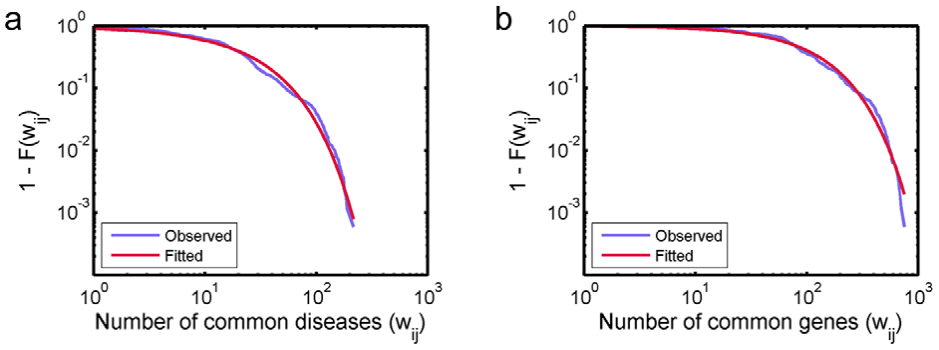
The edge weight distributions from the common disease and gene networks. The distributions of edge weights from the common disease network (a) and the common gene network (b). The complimentary cumulative distribution (1−*F*(*w_ij_*)) of the edge weights (*w_ij_*) as well as the best-fit exponentially-truncated power law curves are plotted.

Brain areas sharing the same diseases or same genes were connected, forming a common disease network and a common gene network, respectively.In these networks, an edge connected two brain areas if a disease or gene influenced both of them.Edge weights in these networks represented the number of diseases or genes shared in common between two brain areas connected (see Figure 3 for the schematic). In the common disease and gene networks, almost all the brain areas are interconnected (Figure 4a and 4b, respectively). However, the strength of connections in these networks, quantified by edge weights, was highly heterogeneous, with a few connections with tremendously large edge weights. This can be seen in the distributions of edge weights in Figure 5 exhibiting characteristics of a long-tail distribution. In fact, more than 2/3 of common diseases or genes can be attributed to the top 30% of all the edges in Figure 5. Althoughvisual inspection of the edge weight distributions suggested theyfollow an exponentially truncated power law distribution, formal statistical tests failed to classify them as exponentially truncated power law distributions (KS test p<0.0001 for both distributions). The implication of this heterogeneity in edge weights is that a small number of brain areas are affected by a large number of the same diseases, or are associated with a large number of same genes. In order to focus on such highly weighted connections, the common disease network and the common gene network were pruned, leaving only the connections with top 10% edge weights. This process resulted in the core networks for the common disease network and the common gene network (Figure 4c and 4d, respectively). These core networks, in a sense, represented the “highways” of the common networks, emphasizing a network of brain regions that are influenced by a large number of the same diseases or genes. The core networks were very similar between the common disease network and the common gene network. This is not surprising since the connections in the common gene network were mediated by disease nodes. In other words, the common gene network shows the number of shared genes among the diseases that affect the same brain areas.Therefore, if two brain areas share a large number of diseases, it is more likely that some of those diseases may share genes in common, resulting in a larger edge weight. If the sharing of genes were limited among the diseases despite the fact that they are connected to the same brain areas, then the resulting common gene network would have a dramatically different pattern of connectivity than that of the common disease network. The brain areas forming these core networks (listed in Table 1) tend to be the high degree brain areas found earlier (see Figure 2a). These areas include those found by recent whole-brain GWAS, including thalamus, cerebellum, hypothalamus [33], and hippocampus [34]–[36]. This is noteworthy because we were able to identify these brain areas based the network data predating these whole-brain GWAS. The gene-disease connection data by Goh et al. [1] precedes both the schizophrenia GWAS [33]and AD GWAS [34]–[36], while the disease-brain connections by PubBrain search precedes the AD GWAS data. Thus, the findings from the core networks demonstrate the utility of the network-based approach as a prediction tool.

**Table 1:** A list of brain areas in the common disease and gene networks.

Core brain areas	Core brain areas
(Common disease network)	(Common gene network)
Basal Ganglia	Basal Ganglia
Caudate nucleus	Caudate nucleus
Globus pallidus	Globus pallidus
Nucleus accumbens	Putamen
Putamen	Striatum
Striatum	Substantianigra
Substantianigra	Brainstem
Brainstem	Medulla oblongata
Locus ceruleus	Midbrain tegmentum
Medulla oblongata	Pons
Midbrain tegmentum	Superior colliculus
Pons	Cerebellum
Cerebellum	Cerebellar cortex
Cerebellar cortex	Diencephalon
Diencephalon	Hypothalamus
Hypothalamus	Pineal body
Pineal body	Thalamus
Thalamus	Frontal
Frontal	Precentralgyrus
Precentralgyrus	Insular
Insular	Insula
Insula	Limbic
Limbic	Amygdala
Amygdala	Hippocampus
Anterior cingulate	White Matter
Hippocampus	Corpus callosum
White Matter	Internal capsule
Corpus callosum	Pyramidal tract
Internal capsule	
Pyramidal tract	

### Disease Connectivity and Potential Biases

**Figure 6:**
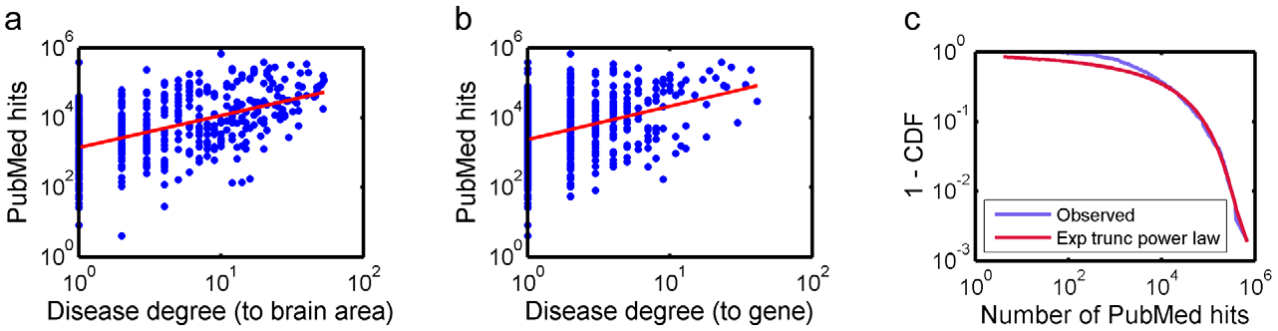
Scatter plots of disease degrees and the number of publications. Disease degrees and the corresponding number of publications are plotted. Disease degrees are for connections to brain areas (a) or genes (b). The number of publications is based on the number of hits on the PubMed database. The correlation was somewhat stronger for the degrees for connections to brain areas (a) (Spearman's correlation ρ = 0.518, p<0.0001) than for connections to genes (b) (ρ = 0.362, p<0.0001). The complementary cumulative distribution (1 - CDF) of the PubMed hits is plotted in (c), along with the best-fit exponentially truncated power law curve. The distribution in (c) indicates that a disproportionately large number of papers (>10,000) have been published on a very few diseases.

**Figure 7:**
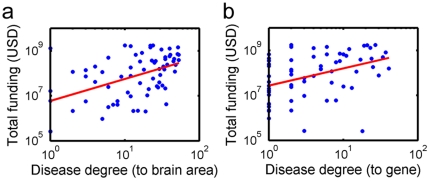
Scatter plots of disease degrees and the amount of research funding. Degrees for a subset of diseases are plotted against the total amount of NIH funding in US dollars (USD) dedicated to those diseases. Disease degrees are for connections to brain areas (a) or genes (b). The amount of NIH funding is based on the RePORTER database. Disease degrees were also correlated with the amount of NIH funding, with somewhat stronger correlation for the degrees for connections to brain areas (a) (Spearman's correlation ρ = 0.480, p<0.0001) than for connections to genes (b) (ρ = 0.436, p<0.0001).

The connections originating from the disease nodes may represent true biological relationships between genes, diseases, and brain areas. Or, they may be biased by research funding for particular diseases or by interests among researchers on particular disorders or conditions. To determine the presence of such biases, we plotted the degree of each disease against the number of publications associated with that disease searched on the PubMed database (see Figure 6). We also plotted the degree of selected diseases against the total research funding by the NIH (National Institute of Health) related to that disease (see Figure 7). Disease degrees were correlated with the number of publications in terms of PubMed hits. The correlation was somewhat stronger for the degrees for connections to brain areas (Figure 6a) (Spearman's correlation ρ = 0.518, p<0.0001) than for connections to genes (Figure 6b) (ρ = 0.362, p<0.0001). These results indicate that diseases with high degrees are likely the highly published ones. It is interesting to note that the distribution of PubMed hits seems to have a long-tail distribution (Figure 6c), indicating tremendous heterogeneity in the number of publications in different genetic disorders, with research activities concentrating on a small number of diseases. Disease degrees were also correlated with the amount of NIH funding, with somewhat stronger correlation for the degrees for connections to brain areas (Figure 7a) (ρ = 0.480, p<0.0001) than for connections to genes (Figure 7b) (ρ = 0.436, p<0.0001). These results show that highly funded diseases tend to be highly connected diseases.

## Discussion

We formed a network of genes, genetic disorders, and brain areas. The resulting network revealed heterogeneity of genetic influence in various brain areas, confirming findings from previous studies that the genetic influence is not uniform in the brain [13], [38]. Moreover, connections in the tripartite network were heterogeneously distributed among nodes. A small number of brain areas, diseases, and genes were connected more abundantly than the vast majority of other nodes. We also examined which different brain areas are affected by the same diseases or associated with the same genes. To this end, a common disease network and a common gene network were generated. These networks revealed a core witha small set of brain areassharing a large number of genes or diseases in common. Thus, these core brain areas are likely affected by many diseases and highly influenced by genes. These brain areas also coincided with the areas indicated in previous whole-brain GWAS [33]–[36]. These findings are particularly interesting since our network approach was able to identify these key brain areas without conducting a large-scale study with explicit hypotheses on gene-brain associations, using the network data that were collected before the publication of the whole-brain GWAS results.

In this study, we were able to generate an overall picture of genetic influences on the brain without focusing on a particular disease or condition. This is in contrast to a conventional imaging genetics study that only allows identification of genes and brain areas connected to a single disease or condition. Although the tripartite network generated in this work is unable to localize significant associations to specific spatial coordinates in the brain or precise loci in the genome, it shows a comprehensive overview of genetic influences on various parts of the brain. This is particularly visible in the common disease and gene networks indicating how a core of brain areas shares the same diseases and genes.

The network-based approach presented here can be an ideal means to consolidate information originating from multiple studies and to identify key components in a system. Although a network of genes, diseases, and/or phenotypes can be constructed from actual assay data (for example, see [3], [4]), such networks can also be formed by connecting nodes based on the knowledge already available in the literature or databases [5], [9], [10], [12]. In other words, networks can be constructed simply by organizing connections discovered by other studies on various populations and conditions. Establishing each of such connections, representing an association between two nodes, may not require a large number of subjects (for example, see [18], [19], [39]). Provided that there is a wealth of literature reporting a large number of connections, constructing and analyzing a network can be done as a secondary analysis without actually collecting data. It is an effective way of using seemingly unrelated information to uncover possibly hidden associations. In fact, the network-based approach has potential as a prediction tool to uncover associations that have not been previously known [2], [3], [40]. Our tripartite network, for example, can be used to identify a group of genetic disorders that affect the same brain areas. By tracing connections originating from this cluster of diseases, one can identify potential associations between genes and brain areas for one of the diseases in the cluster.

Although not all diseases in the OMIM database can be considered as neurological diseases, we feel that no disease should be excluded from the gene-disease-brain tripartite network just because we are not aware of any neurological mechanism associated with seemingly non-neurological diseases. It is true that some neurological symptoms in non-neurological diseases may be coincidental or secondary. However there is also likelihood that such patterns of neurological symptoms may be a result of some neurological pathology we may not be aware of yet. For example, about a decade ago, diabetes was considered an endocrine and metabolic disease, and consequently very few researchers investigated cognitive and neurological implications associated with the disease. Today, on the other hand, cognitive decline and neurological damages associated with diabetes are examined in multiple large-scale epidemiological studies. Likewise there are several studies today examining neurological damages associated with hypertension, of which very little was known just a decade ago. Thus, we believe that excluding non-neurological diseases due to apparent lack of neurological underpinning inhibits the utility and discovery potential of a network-based approach such as ours.

Although this study revealed interesting relationships, there are some limitations associated with our tripartite network. First, connections originating from diseases are highly biased. Diseases that are studied more frequently tend to be connected to a larger number of brain areas or genes. However, it is hard to establish a causal relationship between the number of connections and the number of publications attributed to different diseases. Some diseases may be studied moreextensively than other diseases, resulting in more publications identifying associations. Or, some diseases may have true biological associations with many genes or brain areas, and the large number of publications may be simply a reflection of the large number of such associations. Similarly, it is hard to infer the cause of the strong relationship between the number of connections originating from different diseases and the amount of research funding dedicated to those diseases. In any case, it is clear that the tripartite network generated in this study is likely confounded by other factors such as funding and research activity, and may not purely represent true biological relationships between genes, genetic disorders, and brain areas. We believe that genuine biological effects without any bias could only be identified by constructing a network similar to ours using data deliberately collected for this purpose, as opposed to mining existing data. In other words, a large scale study would be required to identify associations between a large number of genes in multiple disease populations, in which each subject's brain is scanned by different imaging modalities. Conducting such a study is, needless to say, prohibitively expensive and labor intensive, although as brain imaging becomes a more common phenotype in large population-based studies, it may be possible to apply this technique in a relatively unbiased sample in the future. It is true that our network may be biased by non-biological factors, but we believe that a network-based approach such as ours provides a reasonable starting point to focus in future genetic and/or brain imaging studies as long as the biases noted above are taken into consideration.Another limitation is that the database searches were conducted at different time points. The gene-disease connections, extracted from the diseasome network by Goh et al. [1], were gathered prior to 2007, whereas the disease-brain connections were based on the database search conducted in July, 2009. It is likely that the number of diseases in the tripartite network may be smaller than the number of known gene-disease associations today. In addition to the time point of the database queries, the consistency and quality of the search results may also raise a potential problem. For example, some PubMed search results may contain hits from species other than humans, such as non-human primates or rodents. Such non-human hits could occur in a large number in some cases. However, cleaning such results would involve manually verifying each hit and that could be prohibitively labor-intensive. Lastly, in this work, we only considered node degrees and degree distributions to extract relevant information regarding the network. However, other characteristics (modular structure, node centrality, etc) of this network can be examined to reveal additional hidden information in the relationship between genes, diseases, and brain areas.

In conclusion, we constructed a network outlining associations between a large number of genes, genetic disorders, and brain areas. The network was built based on the existing data culled from publicly available databases. The resulting network and characteristics of its connections can reveal relationships between genes, diseases, and brain areas, emphasizing the power of a network-based approach. Moreover, the common disease network and the common gene network revealed the relationship between brain areas that share the same set of diseases or genes. The brain areas constituting the core of these common networks included some areas identified in the previous whole-brain GWAS, and such brain areas will likely be the main focus of future imaging genetics research.

## Methods

### Creating the Tripartite Network

Connections between genes and genetic disorders were based on the network data reported in Goh et al. [1], publicly available on the web. In brief, the connections were made if an association is reported between a genetic disorder and a gene in the OMIM (Online Mendelian Inheritance in Man) database (http://www.ncbi.nlm.nih.gov/omim).

Connections between genetic disorders and brain areas were based on text mining of the PubMed database. The text mining search was interfaced by the PubBrain search engine (http://www.pubbrain.org/). The PubBrain website searches a user-provided search term on the PubMed database together with over 300 brain anatomical terms [37]. In a PubBrain search, if there are any hits (i.e., co-occurrences between the user-provided search term and any of the brain anatomical terms), then the results are presented in the form of a 3D brain heat map, with the intensity representing the number of hits associated with a particular brain area. Along with the heat map, the PubBrain website can also produce a text file listing the number of hits associated with each brain area. PubMed search results in PubBrain are hierarchically organized. This means that hits for a larger anatomical division subsume hits for smaller substructures in that division. For example, hits for the brain stem subsume hits for structures contained in the brain stem, such as the midbrain, pons, and medulla oblongata. Thus the number of hits for the term “brain stem” represents the number of hits for the brain stem as well as the number of hits for the midbrain, pons, and medulla oblongata. The names of all 1284 diseases from the diseasome network were searched using the PubBrain website. In the search results, the brain anatomical terms were reorganized to 65 terms of regions of interest (ROIs) so that the anatomical terms were not too broad (e.g., frontal lobe) or too narrow (e.g., accessory basal amygdaloid nucleus), and the corresponding hits were re-calculated accordingly. A complete list of the 65 brain areas is found in Table S1. A disease and a brain area were considered connected if there were 5 or more hits indicating consistent reporting in the literature. Finally, the disease names were manually re-examined, and duplicate disease names (n = 5) as well as disease names producing erroneous PubMed hits (e.g., Anderson disease, CHILD syndrome, MASS syndrome, etc) (n = 6) were eliminated. These disease names were eliminated because they would produce hits associated with the disease as well as a large number of unrelated results. For example, a search term “Anderson disease” would results in hits associated with the Anderson disease, as well as any papers with an author named “Anderson” discussing a “disease.” Data for the resulting disease-brain network are available in Data S1.

The PubBrain search above resulted in a tripartite network with three layers: genes, genetic disorders, and brain areas. In this network, any nodes without any connections were eliminated. The final tripartite network consisted of 1210 nodes for genes, 509 nodes for genetic disorders, and 59 nodes for brain areas.

### Node Degrees

At each layer of the tripartite network, the number of connections at each node, or degree, denoted by *k*, was examined. In particular, high-degree nodes were identified at each layer. The distribution of the node degree was also examined to assess relative abundance of high or low degree nodes. In many self-organized networks, the degree distribution often follows highly skewed long-tail distributions such as power law distributions [41] or exponentially truncated power law distributions [42]. Such distributions have a long tail spanning multiple orders of magnitude, and often indicate existence of a small number of hubs with extremely high degrees while the vast majority of nodes have just a few connections. While networks with a power law degree distribution have a small number of mega hubs with extremely large degrees, physical or resource constraints in many naturally occurring networks typically inhibit the occurrence of such mega hubs [42]. This limitation often results in a truncated version of a power law degree distribution, such as exponentially truncated power law distributions. In a network with such truncated degree distributions, there are still some hubs with large degrees, but these hubs do not have comparably high degrees as that of mega hubs found in networks with power law degree distributions. For each degree distribution, we fitted an exponentially truncated power law distribution and a power law distribution using the parameter estimation algorithm outlined in Johnson et al. [43]. Even if an observed degree distribution does not follow a particular parametric distribution, such as an exponentially truncated power law distribution or a power law distribution, heterogeneity in node degrees can be easily observed by plotting the degree distribution on a log-log scale. In this study, complimentary cumulative distributions (1−F(k)) were plotted on a log-log scale; this way, the y-coordinate of a point on an observed distribution curve can be interpreted as the empirical p-value for the node degree corresponding to the x-coordinate. If there are a small number of high degree nodes, often at least one order of magnitude larger than the majority of other nodes, then such nodes can be easily identified at the tail of the distribution curve.

The goodness-of-fit for an observed degree distribution can be assessed statistically, for example by a Kormogorov-Smirnov (KS) test. However, a KS test may not be uniformly sensitive over the wide range of degrees covered in a typical long-tail degree distribution. Even if the tails of the observed and theoretical distributions may appear close to each other on a log-log scale, a KS test statistic is likely dominated by the difference between the observed and theoretical distributions among low degree nodes. This is because the difference between the observed and theoretical is a few orders of magnitude smaller near the tail of the degree distribution compared to the difference near the lower end of the degree range. Although we provide p-values based on KS tests for the observed degree distributions from the tripartite graph by comparing them to exponentially truncated power law and power law distributions, lack of good fit does not imply a lack of heterogeneity in node degrees. The instability of the KS test statistic near the tail of long-tailed distributions was noted by Stumpf et al. [44], and they suggested the Anderson-Darling (AD) test as an alternative approach to the KS test. The AD test is sensitive near the tails of the null distribution in contrast to the KS test which is sensitive near the median [45]. Unfortunately critical values of the AD test are dependent on the null distribution. It is possible to obtain critical values numerically for exponentially truncated power law distributions and power law distributions by a simulation-based approach [44], but performing such simulations is beyond the scope of this paper.

### Collapsing the Tripartite Network

Among the brain areas represented in the tripartite network, some may be affected by the same set of diseases, or may be associated with the same set of genes. To understand how brain areas share the same connections to diseases or genes, the tripartite network was collapsed as a set of connections between brain areas representing shared genes or diseases. In particular, a common disease network and a common gene network were constructed. The common disease network was organized by connecting two brain areas that are connected to the same diseases (see Figure 3, left). The number of shared diseases between two brain areas was used as the edge weight between them. Common diseases between all pairs of brain areas were examined, resulting in a network of brain areas connected by shared diseases. The common gene network was constructed upon the same principle as the common disease network, except for the fact that the connections were made on the basis of shared genes between two brain areas (see Figure 3, right). Since genes were not directly connected to brain areas, shared genes between two brain areas were mediated by diseases. The edge weight in the common gene network represented the number of genes shared in common between brain areas.

In the common disease network and the common gene network, edge weights were further examined in order to identify brain areas sharing a large number of diseases or genes. To do so, first the distribution of edge weights was examined to identify any heterogeneity in the number of shared diseases or genes among the brain areas. We also formed the core networks, the networks of brain areas formed by the connections with top 10% of edge weights in the common disease and gene networks, accentuating the commonality among the brain areas sharing the same diseases or genes.

One of the goals of the core networks is to extract meaningful information from the common disease and gene networks, which are almost fully connected (see Figure 4a and 4b). Another goal of the core networks is to show how some brain areas are affected by a large number of the same diseases. For example, both the hippocampus and the corpus callosum are associated with a large number of diseases (258 and 213, respectively, see Figure 2a), but this does not imply that these areas are associated with the same set of diseases. In other words, diseases that affect the hippocampus could be a completely different type of diseases than the ones affecting the corpus callosum. The core networks allow us to ascertain and visualize whether different brain areas indeed do share the same diseases or genes. This information cannot be obtained by simply examining the high-degree brain-area nodes individually. Moreover, strong links between different brain areas may indicate consistent co-occurrence of pathological processes on those brain areas.

### Examining Biases

Gene-disease connections and disease-brain connections in the tripartite network were based on the data from publicly available databases. However, the data in those databases may be biased toward diseases that are highly studied among researchers. For example, some diseases may be investigated more often than other diseases. Or some diseases may be studied more widely due to favorable availability of research funding for those diseases. To examine such potential biases, the diseases from the tripartite networks were queried in PubMed to gauge the research activity associated with those diseases as the number of publications. The number of PubMed hits and the degrees for the corresponding diseases were plotted to assess any association. In addition, a potential funding bias was investigated among 69 diseases selected from the tripartite network to cover the entire range of disease degrees. For each of these diseases, the amount of total research funding was searched on the RePORTER (Research Portfolio Online Reporting Tools Expenditures and Results) database, a publicly available database of research funding by the NIH (National Institute of Health) of the United States. The amount of total NIH funding and the disease degree were plotted to assess any association between them.

## Supporting Information

Table S1 **A list of 65 brain anatomy terms used in the PubBrain searches.**
(DOCX)

Data S1 **A node list and an edge list of the disease-brain network in Pajek format.**
(NET)
