# Supplementary material for: A Network of Genes, Genetic Disorders, and Brain Areas
Source: PLoS One. 2011 Jun 10;6(6):e20907. doi: 10.1371/journal.pone.0020907 (PMC3112220; doi:10.1371/journal.pone.0020907)
Supplement: Table S1 — A list of 65 brain anatomy terms used in the PubBrain searches. (DOCX) [file pone.0020907.s001.docx]

***Table S1****: A list of 65 brain anatomy terms used in the PubBrain searches*

| Class | Brain areas |
| --- | --- |
| Basal Ganglia | Caudate nucleus |
|  | Globus pallidus |
|  | Nucleus accumbens |
|  | Nucleus lentiformis |
|  | Putamen |
|  | Striatum |
|  | Substantia nigra |
|  | Subthalamic nucleus |
| Brainstem | Inferior cerebellar peduncle |
|  | Inferior colliculus |
|  | Lateral geniculate body |
|  | Locus ceruleus |
|  | Medial geniculate body |
|  | Medulla oblongata |
|  | Midbrain reticular formation |
|  | Midbrain tegmentum |
|  | Pons |
|  | Red nucleus |
|  | Superior cerebellar peduncle |
|  | Superior colliculus |
|  | Tectum |
| Cerebellum | Cerebellar cortex |
|  | Cerebellar nuclear complex |
| Diencephalon | Hypothalamus |
|  | Pineal body |
|  | Thalamus |
| Frontal | Inferior frontal gyrus |
|  | Lateral orbital gyrus |
|  | Medial orbital gyrus |
|  | Middle frontal gyrus |
|  | Orbitofrontal cortex |
|  | Posterior orbital gyrus |
|  | Precentral gyrus |
|  | Straight gyrus |
|  | Superior frontal gyrus |

***Table S1*** *(Continued)*

| Class | Brain areas |
| --- | --- |
| Insular | Insula |
|  | Opercular cortex |
| Limbic | Amygdala |
|  | Anterior cingulate |
|  | Hippocampus |
|  | Parahippocampal gyrus |
|  | Posterior cingulate |
| Occipital | Cuneus |
|  | Lateral occipital gyrus |
|  | Lingual gyrus |
|  | Superior occipital gyrus |
| Parietal | Angular gyrus |
|  | Postcentral gyrus |
|  | Precuneus |
|  | Superior parietal lobule |
|  | Supramarginal gyrus |
| Temporal | Fusiform gyrus |
|  | Heschl's gyrus |
|  | Inferior temporal gyrus |
|  | Middle temporal gyrus |
|  | Planum temporale |
|  | Superior temporal gyrus |
| White Matter | Arcuate fasciculus |
|  | Corpus callosum |
|  | Inferior longitudinal fasciculus |
|  | Inferior occipitofrontal fasciculus |
|  | Internal capsule |
|  | Pyramidal tract |
|  | Superior longitudinal fasciculus |
|  | Superior occipitofrontal fasciculus |
